# Massive Hemoptysis in Pulmonary Tuberculosis From Rasmussen Pseudoaneurysm

**DOI:** 10.7759/cureus.30117

**Published:** 2022-10-10

**Authors:** Ahmed Shebani, Mohamed Hnish, Hussam Elmelliti, Ahmed Lutfe Abdussalam

**Affiliations:** 1 Internal Medicine, Hamad Medical Corporation, Doha, QAT; 2 Family Medicine, Hamad Medical Corporation, Doha, QAT; 3 Emergency Medicine, Hamad Medical Corporation, Doha, QAT; 4 Critical Care, Hamad Medical Corporation, Doha, QAT

**Keywords:** tuberculosis, medical icu, massive hemoptysis, rasmussen’s aneurysm, respiratory complications

## Abstract

Massive hemoptysis due to pulmonary tuberculosis is a life-threatening complication; it occurs as a result of the erosion of one of the pulmonary vessels. Tuberculous vascular lesions can also lead to arteritis, thrombosis, arterial dilation and Rasmussen aneurysms. "Rasmussen aneurysm" is a rare cause of hemoptysis. The extent of hemoptysis varies in severity from mild to life threatening, which is more common.

Here, we report a case of a 45-year-old Indian male who initially presented with cough, and generalized weakness; his clinical, laboratory, and radiological findings were highly suggestive of pulmonary tuberculosis. Following medical ward admission, two weeks later he had worsening of his respiratory status complicated by massive hemoptysis and dropped oxygen saturation requiring intubation and admission to the intensive care unit. His computed tomography angiography revealed localized aneurysmal dilatation of the pulmonary artery in the left lower lobe (Rasmussen aneurysm); embolization was performed successfully. Due to the poor respiratory reservoir in most pulmonary TB cases, interventional radiology is preferred over surgery.

## Introduction

As one of the top 10 global causes of death, tuberculosis is a contagious disease that is a significant contributor to poor health. It is also the leading infectious agent-related cause of death, surpassing HIV/AIDS. *Mycobacterium tuberculosis*, a species of pathogenic bacteria, is what causes tuberculosis; although tuberculosis primarily affects the lungs, the illness can also affect other locations (extrapulmonary tuberculosis). A quarter of the world's population has *M. tuberculosis* infection. Geographically, according to the 2020 WHO report, the highest rates of tuberculosis cases in 2019 were found in South-East Asia (44%), Africa (25%), and the Western Pacific (18%); lower rates were found in the Eastern Mediterranean (8.2%), the Americas (2.9%), and Europe (2.5%). A total of eight nations, namely, India (26%), Indonesia (8.5%), China (8.4%), the Philippines (6%), Pakistan (5.7%), Nigeria (4.4%), Bangladesh (3.6%), and South Africa (3.6%), accounted for two-thirds of the global total. The remaining 22 nations on the WHO's list of 30 nations with a high tuberculosis burden accounted for 21% of the global total [1].

Hemoptysis, pneumothorax, bronchiectasis, extensive pulmonary destruction (such as pulmonary gangrene), fistula, tracheobronchial stenosis, malignancy, and chronic pulmonary aspergillosis are all complications of pulmonary tuberculosis. Massive hemoptysis has been found to be responsible for roughly 5% of tuberculosis deaths prior to effective treatment [2,3]. Massive hemoptysis in pulmonary tuberculosis comes from the erosion of vessels in pulmonary circulation, and intercostal arteries.

Tuberculous vascular lesions can include pulmonary or bronchial arteritis and thrombosis, as well as bronchial artery dilatation (Rasmussen aneurysm). The term "Rasmussen aneurysm" refers to the formation of an aneurysm as a result of a cavitary infection that spreads into the adventitia and media of bronchial arteries, causing inflammation and thinning of the vessel wall [4,5]. This aneurysm then ruptures into the cavity, resulting in massive hemoptysis. The incidence of Rasmussen aneurysm is very rare, and the extent of hemoptysis varies in severity from mild to life threatening as reported in various case reports. One case from Saudi Arabia, in 2018, of pseudoaneurysm also presented with massive hemoptysis, which was treated with embolization and coil insertion; unfortunately, the patient passed away despite all the measures [6]. This highlights the severity of massive hemoptysis caused by Rasmussen aneurysm. On the other hand, there was another case from India in 2020 that had a more fortunate course, as it only presented with mild hemoptysis and did not progress further [7].

## Case presentation

**Figure 1:**
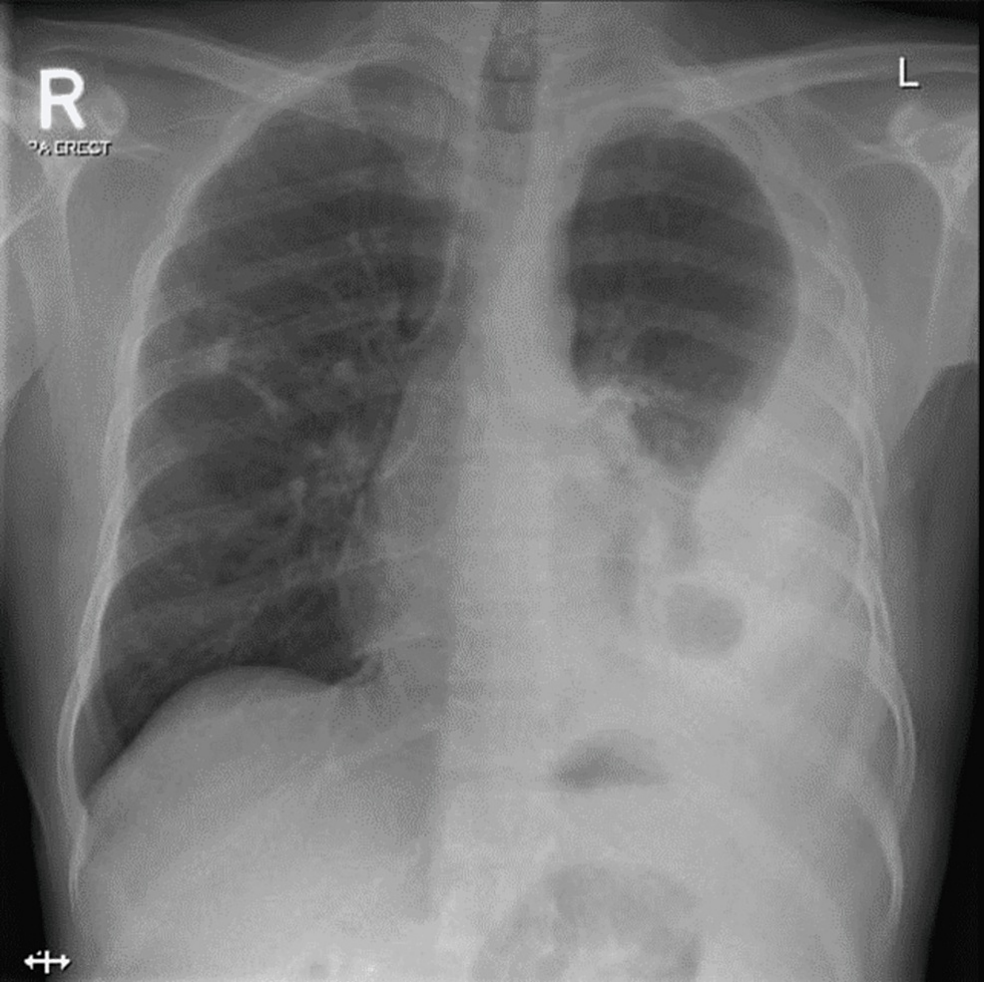
Chest X-ray showing large left-sided pleural effusion with collapse-consolidation of the left lung lower lobe. A patchy nodular opacity is seen in the right lung mid-zone

A 45-year-old Indian male, with type 2 diabetes mellitus and chronic kidney disease, was admitted to the medical ward as a case of pulmonary tuberculosis based on a positive acid-fast bacilli sputum sample and a chest X-ray finding of a large left-sided pleural effusion with collapsed consolidation of the left lung lower lobe. A patchy nodular opacity was seen in the right lung mid-zone (Figure 1). On day 10 of admission, the patient developed acute massive hemoptysis along with desaturation, hypotension and decreased level of consciousness. After transfer to the intensive care unit, he became increasingly hypoxic and unresponsive. His oxygen saturation dropped to <70%; heart rate was 150 beats/minute and blood pressure was 160/90 mmHg. The patient was intubated and started on mechanical ventilator; fresh blood was noticed from the endotracheal tube. Saturation improved to 96%; his baseline hemoglobin (Hb) level was 12.7 g/dl (normal 13-17 g/dl) that dropped to 11 g/dl. He received two units of packed RBC transfusion, but the Hb level continued to drop to reach 9 g/dl. An immediate bedside bronchoscopy showed an active bleeding from the left main bronchus; the field was full of blood. He started to desaturate, so the procedure was aborted; the patient was sent for pulmonary computed tomography angiography (CTA) after he was stabilized.

**Figure 2:**
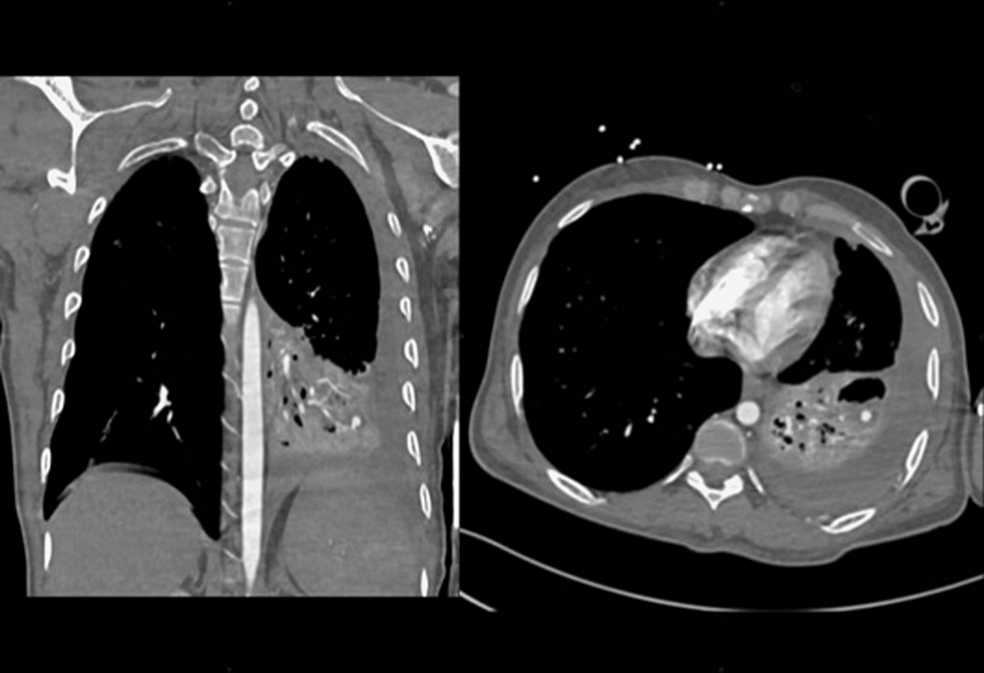
The CT pulmonary angiogram showing left lower lobe near complete collapse-consolidation with at least two cavitary lesions. Small pseudoaneurysm is noted measuring 5 x 6.6 x 5 mm arising from a subsegmental branch of the left lateral basal segmental artery of the left lower lobe adjacent to the cavitary lesion (Rasmussen aneurysm) in the background of pulmonary tuberculosis.

A CT pulmonary angiogram showed the left lower lobe near complete collapse-consolidation with at least two cavitary lesions. A small pseudoaneurysm was noted measuring 5 x 6.6 x 5 mm arising from a subsegmental branch of the left lateral basal segmental artery of the left lower lobe adjacent to the cavitary lesion (Rasmussen aneurysm) (Figure 2).

The interventional radiology team was consulted, and an angiography was performed on the left pulmonary artery, with a small pseudoaneurysm arising from one of the inferior segmental distal branches. Embolization was performed, and completion angiogram showed the disappearance of the pseudoaneurysm. The patient did not have any further episodes of hemoptysis or bleeding and was extubated two days later. He was then moved to a regular medical floor and was discharged a few days later on full anti-tuberculosis chemotherapy regimen.

## Discussion

An aneurysm is defined as a focal dilatation of all three layers of the pulmonary artery wall whereas a pseudoaneurysm does not involve all three layers. Despite the difference, the terms are interchangeable in the literature [8]. In pulmonary tuberculosis, hemoptysis can occur because of bronchiectasis, more commonly, erosion in the bronchial artery, or rarely, from the pulmonary artery, which is also known as Rasmussen aneurysm, a complication first described by Fritz Waldemar Rasmussen in 1868 [9]. The term "Rasmussen aneurysm" is uniquely reserved to tuberculous etiology. This phenomenon occurs when the tuberculous cavitary lesion erodes into adjacent pulmonary structures; a branch of the pulmonary artery wall weakens as the tuberculous granulation tissue invades both the adventitia and the media layers with fibrin deposition. Afterwards, the arterial wall progressively becomes thinner that leads to pseudoaneurysm formation. These pseudoaneurysms can potentially rupture, resulting in life-threatening massive hemoptysis.

In a study, patients with confirmed pulmonary tuberculosis and hemoptysis were referred for multidetector CT (MDCT) scanning with pulmonary angiography. Rasmussen aneurysms were found in only 54 cases (0.25%) and Rasmussen pseudoaneurysm in only 3 cases (0.01%) [10].^ ^However, the incidence of pulmonary tuberculosis with cavitary lesions presenting with massive hemoptysis is higher, as evidenced by eight patients with severe hemoptysis uncontrolled by previous bronchial and systemic arterial embolization.

In the management of massive hemoptysis, the priority is securing the airway, maintaining oxygenation and ventilation. The role of flexible fiberoptic bronchoscopy is paramount, as it is available at bedside and has high diagnostic and therapeutic benefits [11]. In our case, bedside bronchoscopy showed active oozing of blood from the left main bronchus, but the source of the bleeding could not be identified. When bronchoscopy is not available or cannot reveal the source of bleeding, the pulmonary CT angiography scan is used as an alternative diagnostic option because of its high sensitivity in guiding the therapeutic intervention. Clinical studies have shown that CT scans are the most sensitive diagnostic tool, especially when combined with bronchoscopy, as was done in our case [12].

To achieve hemostasis in the patient, the merits of interventional radiology modalities versus surgical options are considered. Although evidence comparing these two options is lacking, many centers initially prefer minimally invasive approaches. Thus, interventional radiology is the preferred option due to the poor respiratory reservoir in most pulmonary TB cases, which renders them poor surgery candidates.

Angiographic embolization in massive hemoptysis has been reported to achieve a more than 90% success rate in controlling all causes of massive hemoptysis [13]. Bronchial artery embolization of massive hemoptysis constitutes the majority of published literature, and only a few cases of successful endovascular embolization of pulmonary artery pseudoaneurysms with massive hemoptysis have been described. Our case represents successful left pulmonary artery angiography, supra-selective embolization and coiling of the inferior segmental pulmonary artery pseudoaneurysm and complete control of the hemoptysis.

## Conclusions

In pulmonary tuberculosis, hemoptysis can occur because of bronchiectasis, more commonly, erosion in the bronchial artery, or rarely from the pulmonary artery, which is also known as Rasmussen aneurysm. The priority in the treatment of massive hemoptysis is to secure the airway, maintain oxygenation and ventilation. The role of flexible fiberoptic bronchoscopy is critical because it is available at the patient's bedside and has a high diagnostic and therapeutic benefit. Pulmonary CT angiography scans are used as an alternative diagnostic option when bronchoscopy is not available or cannot reveal the source of the bleeding. Due to the poor respiratory reservoir in most pulmonary TB cases, interventional radiology is preferred over surgery. Our case represents a successful left pulmonary artery angiography, supra-selective embolization and coiling of the inferior segmental pulmonary artery pseudoaneurysm, as well as complete hemoptysis control.
